# Prevalence of Work-Related Pain or Discomfort Among Urologists in the State of Florida: Results From the Florida Urologic Society Task Force on Ergonomic Challenges Experienced by Its Members

**DOI:** 10.2196/88848

**Published:** 2026-06-17

**Authors:** Justin David, Austin Hill, Jared Schommer, Eric Regele, Colleen Ball, Sanoj Punnen, Joseph Costa, Adam Ball, Jamin Brahmbhatt, David D Thiel, Raymond Pak, Ram A Pathak

**Affiliations:** 1 Mayo Clinic in Florida, 4500 San Pablo Rd S, Jacksonville, FL, 32224, United States, 1 2255052210; 2 Mayo Clinic, Rochester, MN, United States; 3 Desai Sethi Urology Institute, Miami, FL, United States; 4 University of Florida Health- Jacksonville, Jacksonville, FL, United States; 5 Gulf Stream Urology Associates, Port St. Lucie, FL, United States; 6 Orlando Health, Orlando, FL, United States

**Keywords:** ergonomics, robotics, laparoscopy, endoscopy, urology, pain

## Abstract

**Background:**

Pain and work-related musculoskeletal disorders are commonly seen in surgeons, significantly impacting quality of life and burnout. A questionnaire-based study was conducted to further investigate the nature and etiology of work-related pain among urologists in the state of Florida.

**Objective:**

This study aimed to quantify the number of urologists who reported work-related musculoskeletal disorders >25% of the time.

**Methods:**

The Florida Urologic Society Task Force developed a survey based on the Nordic Musculoskeletal Questionnaire, with additional input from Cornell’s ergonomic studies. The Mayo Clinic Survey Research Center conducted the survey and distributed it to 504 members of the Florida Urologic Society in 2023.

**Results:**

The total response rate was 18.6% (94/504). The primary outcome (number of urologists who reported pain >25% of the time) was 45.3% (34/75). In total, 32.4% (22/68) of the respondents reported pain associated with endoscopic surgery >25% of the time, 40.0% (14/35) reported pain for major open cases, 20.6% (13/63) reported pain for minor open cases, and 22.7% (5/22) reported pain for robotic cases. In total, 68.8% (53/77) of the respondents attributed their work-related pain to uncomfortable operating positions, and 29.9% (23/77) chose to ignore their pain.

**Conclusions:**

In this contemporaneous population of Florida urologic surgeons, almost half of the respondents describe having work-related pain >25% of the time. The data show that major open surgery had the highest rate of pain, followed closely by endoscopic surgery. Over 70% of the urologists in Florida are interested in official ergonomics training, which, if developed, may lead to increased productivity and better emotional, personal, and interpersonal well-being.

## Introduction

In recent years, ergonomics has gained considerable interest due to the increasing conscientiousness for physical and mental well-being [1-4], including in the field of urology. In the era of open pelvic surgery, complaints of work-related musculoskeletal disorders (WRMDs) were very common, present in more than 66% of surgeons [5,6]. Despite the invention of minimally invasive techniques such as laparoscopic or endoscopic surgery, WRMDs are just as common [5]. A study reported that urologists who regularly perform endoscopic procedures are at a 3 times higher risk for developing a chronic musculoskeletal disorder than those who do not [2].

However, even with attempts at improving ergonomics, such as robotic surgery, WRMDs are still present. A survey of robotic surgeons showed that pain is very prevalent, with the neck (73.7%), shoulder (52.6%), and lower back (42.1%) being the most common areas [1]. An astounding 70% of urologists performing robotic, open, or endoscopic cases have used nonsteroidal anti-inflammatory drugs to cope with musculoskeletal pain while operating [3]. The type of injury likely differs. For example, WRMDs associated with major open surgery may be more related to the trunk and neck, while robotic surgeons have higher hand and/or wrist injuries [1,5,6]. Both open and robotic surgery have seen improvements with respect to ergonomics. Prismatic loupes and passive neck exoskeletons are recent improvements for open surgery that have been shown to decrease pain [7,8]. Robotic consoles continue to improve, such as the da Vinci 5 allowing for straighter back and neck posture and lighter hand controls with a rubber grip to improve finger or wrist discomfort [9].

Robotics and endoscopy are a significant part of many urologists’ surgical schedules; however, 80% report no formal ergonomic training in the operating room [3]. This research indicates that there is a high prevalence of work-related pain experienced by surgeons and that minimal ergonomic education has taken place to prevent such problems.

In conjunction with the Florida Urologic Society (FUS), we sent a survey to urologists in the state of Florida to gather more contemporary information regarding the prevalence of work-related pain and discomfort to better understand the role ergonomics plays among Florida urologists.

## Methods

### Overview

The FUS funded and conducted this study in partnership with the Mayo Clinic Survey Research Center. A survey was developed based on the Nordic Musculoskeletal Questionnaire with additional input from Cornell’s ergonomic studies, which is provided in Multimedia Appendix 1. This survey contained 24 multiple choice questions. There are 10 questions used to delineate the etiology, frequency, and treatment of pain. The next 8 questions inquire about the types of surgeries performed and the pain associated with them. A total of 2 questions assess if surgeons received ergonomic training or if they have interest in training. The final 4 questions consist of the surgeons’ demographics and years spent in practice. The Mayo Clinic Survey Research Center distributed the survey to members of the FUS consisting of 504 practicing urologists in 2023.

The primary outcome was the number of urologists who reported pain >25% of the time during surgery. Secondary outcomes included demographics and the type of procedure, length of the procedure, cause of discomfort, specific muscles affected, and methods attempted to minimize discomfort. Inclusion criteria included members of the FUS in 2023, and there were no exclusion criteria. Data collection was performed using Research Electronic Data Capture (REDCap; Vanderbilt University) software, and the number of each response out of the total number of responders for each question was recorded. The total number of respondents was identified for each question, and the number of answers for each option was added. Sample size is dependent on response rate.

### Ethical Considerations

This study was exempt from institutional review board approval.

## Results

### Demographics

In total, 18.6% (94/504) of the urologists within the FUS returned the survey. Not all participants completed every question.

Demographic data are presented in Table 1. In total, 46.8% (44/94) of the respondents worked in private practice, 30.9% (n=29) worked at a teaching-based hospital with resident assistance, 9.6% (n=9) worked at a teaching-based hospital without resident assistance, and 12.8% (n=12) worked at a community hospital.

**Table 1.** The type of urology practice was selected in the survey, with percentages of each answer provided in the table.

Type of urology practice	Values, n (%)
Private practice	44 (47)
Teaching-based hospital with resident assistance	29 (31)
Community hospital	23 (13)
Teaching-based hospital without resident assistance	9 (9)

In total, 76.6% (72/94) of the respondents completed the question regarding their experience level. Of the 72 respondents, 5.6% (n=4) were residents, 12.5% (n=9) had been in practice less than 5 years, 30.6% (n=22) had been in practice for 6 to 20 years, and 51.3% (n=37) have been practicing for more than 20 years.

In total, 75.5% (71/94) of the respondents completed the question regarding their gender identity, height, and weight. Of the 71 respondents, 84.5% (n=60) were male, 11.2% (n=8) were female, 2.8% (n=2) chose another description, and 1.4% (n=1) chose not to answer. Respondents could also choose their height and weight in a range. The most common height was between 5’9” to 6’0” (n=27, 38.0%) followed by 5’5” to 5’8” (n=25, 35.2%). The most common weight range was 151 to 200 lbs (n=47, 66.2%).

### Types of Surgeries Performed: Endoscopic (Question 9)

In total, 79.7% (75/94) of the respondents completed the questions regarding type of surgery performed. Of the 75 respondents, 33.3% (n=25) performed between 1 and 5 endoscopic cases weekly, while 57.3% (n=43) performed >5 weekly.

### Types of Surgeries Performed: Open (Question 11 and 13)

In total, 45.3% (34/75) of the respondents performed 1 to 4 major open cases, defined as cases taking more than 3 hours, while 53.3% (n=40) performed 0 weekly, and 1.3% (n=1) performed >4 cases. In total, 66.7% (n=50) of the respondents performed 1 to 4 minor open cases, 14.6% (n=11) performed 0, and 17.3% (n=13) performed >4 cases.

### Types of Surgeries Performed: Robotic (Question 15)

In total, 70.7% (53/75) of the respondents performed 0 robotic cases weekly, while 22.7% (n=17) performed 1% to 3%, and 6.7% (n=5) performed >3 robotic cases per week.

### Etiology of Pain or Discomfort

In total, 88.3% (83/94) of the respondents completed the question regarding types of WRMDs. Of the 83 respondents, 90.4% (n=75) reported pain at any point in the last week.

The primary outcome was pain or discomfort greater than 25% of the time during surgery. In total, 79.8% (75/94) of the respondents answered questions regarding the frequency of pain depending on the type of surgery. Of the 75 respondents, 45.3% (n=34) reported pain or discomfort greater than 25% of the time during surgery. Years in practice was not a significant predictor of pain, as 53.8% (7/13) of the urologists in practice for 5 years or less reported pain greater than 25% of the time during surgery, while 45.9% (17/37) of the urologists in practice for more than 20 years reported pain greater than 25% of the time during surgery.

In total, 32.8% (22/68) of the urologists who perform endoscopic surgery reported pain or discomfort for at least 25% of the time. In total, 32.0% (8/25) of the urologists who perform less than 5 endoscopic cases per week reported pain, while 32.6% (14/43) who perform greater than 5 cases reported pain. In total, 40.0% (14/35) of the urologists who perform major open surgery reported pain, with 41.8% (14/34) who perform 1 to 4 cases per week reported pain and 0% (0/1) of urologists who perform more than 4 major open cases per week. In total, 20.6% (13/63) of the urologists who perform minor open cases reported pain, with 14.0% (14/50) in those who perform 1 to 4 cases per week and 46.2% (6/13) in those who perform more than 4 cases. In total, 22.7% (5/22) of the urologists who perform robotic cases reported pain or discomfort at least 25% of the time, with 11.8% (2/17) in those who perform 1 to 3 cases per week and 60.0% (3/5) in those who perform more than 3 robotic cases per week.

We then analyzed the cause of discomfort (Table 2). In total, 18.2% (14/77) of the respondents reported no work-related discomfort, while 68.8% (n=53) of the respondents attributed their work-related pain and discomfort to an uncomfortable operating position, 51.9% (n=40) to extensive standing, 35.1% (n=27) to equipment hindrance, 19.5% (n=15) to heavy tools, 15.6% (n=12) to moving patients, and 10.4% (n=8) to another reason not listed on the survey.

**Table 2.** The causes of work-related pain, with the percentage of each response detailed in the right-hand column.

Cause of work-related pain	Values, n (%)
No work-related pain/discomfort	14 (18)
Uncomfortable operating position	53 (69)
Extensive standing	40 (52)
Equipment hindrance	27 (35)
Heavy tools	15 (20)
Moving patients	12 (16)
Other	8 (10)

### Surgeon-Directed Measures to Reduce Pain or Discomfort

In total, 61.0% (47/77) of the respondents changed positions, 37.7% (n=29) used a chair for seated surgery, 35.1% (n=27) implemented wearing supportive footwear or stockings, and 31.2% (n=24) took microbreaks. Furthermore, 29.9% (n=23) of the respondents chose to ignore their intraoperative discomfort, 37.7% (n=29) managed their pain with over-the-counter medications, 14.3% (n=11) pursued medical attention, 14.3% (n=11) decreased surgical workload, and 10.4% (n=8) took time away from the operating room. In total, 6.49% (n=5) of the respondents chose to change to robotic surgery to attempt to alleviate pain.

The survey also explored the types of neuromusculoskeletal disorders formally diagnosed by the surgeon’s medical care team, which revealed that 17.1% (13/76) of the respondents had cervical disc disease, 7.9% (n=6) reported wrist or forearm tendonitis, and 28.9% (n=22) reported back pain secondary to lumbar disc disease. In total, 46.6% (35/75) of the respondents stated that their sleep was affected by their work-related discomfort, 45.3% (n=34) stated that their work-related pain affected their surgical posture, 22.7% (n=17) reported deleterious effects on their mobility, and 21.3% (n=16) reported decreased stamina.

We then studied pursued medical intervention. In total, 35.1% (26/74) of the respondents pursued massage therapy, 27.0% (n=20) required diagnostic studies, 24.3% (n=18) required prescription medications, 20.3% (n=15) required referral to a specialist, 18.9% (n=13) used a brace or support device in the operating room, and 16.2% (n=12) even needed surgical intervention (Table 3).

Finally, 75% (54/72) of respondents have never received formal ergonomic training, and 72% (n=52) would be interested in completing such training.

**Table 3.** Methods used to minimize operative discomfort. Respondents could select multiple options.

Methods to minimize operative discomfort	Values, n (%)
Seek medical help	11 (14)
Changing position	47 (61)
Take a break	24 (31)
Go slower	3 (4)
Adjust some aspect of the surgical field	26 (34)
Change height by using step	23 (30)
Use chair for seated surgery	29 (38)
Changing instruments	4 (5)
Switch to robotic surgery	5 (6)
Consider different operative approach	5 (6)
Reduce case load	11 (14)
Footwear or support stockings	27 (35)
Surgical floor mats	16 (21)
Time away from the operating room	8 (10)
Over-the-counter medication	29 (38)
Ignore it	23 (30)
Other	8 (10)

## Discussion

### Principal Findings

The results of the present survey showed the prevalence of WRMDs in Florida urologists estimated at 45.3%, which is consistent with the literature estimating around 47% to 62% prevalence for urologists, who are at risk due to the high use of minimally invasive surgical modalities such as endoscopy, laparoscopy, and robotics [2]. Our definition of WRMD included those who experienced pain at least 25% of the time during surgery, while comparative studies have less restrictive criteria.

Our survey not only provided more information concerning the prevalence of WRMDs and lack of ergonomic education within the field of urology but also provided valuable details about which types of cases are causing injury, the circumstances during those cases causing discomfort, methods being used to combat this issue, and how these injuries are affecting physician performance. In total, 80% (75/94) of the respondents described having pain in the workplace, primarily due to an uncomfortable operating position or extensive standing.

Over half of the survey respondents reported being in practice for a minimum of 20 years, raising the possibility of selection bias. Further review of this cohort of surgeons revealed that almost half of the respondents performed open surgery weekly, which likely influenced the nature of WRMDs. While the number of years in practice did not influence prevalence of pain, the number of surgeries performed per week, especially for minor open cases and robotics, may influence the prevalence of WRMDs.

An intentional byproduct of robotic surgery was to lessen the ergonomic demand of surgeons. However, WRMDs associated with robotic surgery are still prevalent, but the affected body part may differ. In our study, the prevalence of WRMDs was higher with major open (14/35, 40.0%) versus robotic surgery (5/22, 22.7%)

Our survey found that over 1 in 4 urologists sought medical care for their pain, which contributes substantially to health care costs as the median estimated cost per claim for WRMDs is over US $20,000 [10]. While many urologists attempted to treat their pain with over-the-counter medications or other modalities, almost a third of respondents ignored their pain. Ignoring the pain is an alarming finding, as it often coexists with physician burnout [11].

A significant takeaway is the disparity between infrequency, effectiveness, and substantial interest in ergonomic training. Our study showed that 75% (54/72) of the urologists had no formal training, and 72% (52/72) would be interested in such education. A study evaluating the effectiveness of ergonomic training showed that 100% (32/32) of those who received such instruction found it helpful, 88% (28/32) changed their practice as a result, and 74% (24/32) noticed a decrease in strain after training [1]. This particular study also showed that a small time commitment to ergonomic training can dramatically decrease the incidence of strain and injury from performing robotic procedures by greater than 70% [1]. Because of the effectiveness of ergonomics training, its implementation into practice may improve the pain and discomfort experienced during urologic surgery. If actual training sessions are not possible, general advice regarding acceptable intraoperative modifications to prevent injury should be practiced more routinely. Some tips include modifying table height to decrease back strain, maintaining a neutral position throughout a procedure, and taking 2-minute pauses every half hour or as needed to move, stretch, and relax during cases and stretching between cases [12-15]. Regarding robotics specifically, it has been associated with surgeon discomfort especially in the neck and trunk [16]. Becoming familiar with the robot’s ergonomic settings has been shown to lower the rate of physical symptoms during and after procedures by not allowing greater than 20 degrees of neck flexion, avoiding excess knee flexion, and using the clutch often to restore proper and comfortable posture frequently [15].

There are limitations to this study. Any questionnaire-based study is associated with selection bias, as participants who complete the study may be more likely to have physical ailments or WRMDs. In total, 18.6% (94/504) of the recipients completed the survey, which is near the response rate of similar studies assessing surgical WRMDs [17-19]. Despite the consistent response rate with other studies, the low response rate may not truly represent the burden of WRMDs. A significant number of respondents practiced >20 years, risking the potential of selection bias. This study is also limited by convenience sampling as only urologists from Florida were included and respondents may have been affected by their accessibility to the survey. Another limitation is the inability to account for other confounding variables outside a surgeon’s career, such as preexisting conditions or injuries that could contribute to pain. Additionally, many urologists perform several different types of surgeries, but it may be difficult to attribute their pain to a particular type. Our survey could not account for the type of practice a urologist has, as surgeons in an academic setting may have resident substitutes for parts of the surgery.

The goal of this survey was to explore the prevalence and etiology of pain in urologists. With further knowledge about the etiology of urologic surgery–related pain in this contemporary cohort, we hope that changes can be implemented to minimize the pain and improve work-related satisfaction, comfort, and surgeon longevity.

### Conclusions

Surgeons are burdened by WRMDs far too frequently. Our survey found that nearly half of the urologists who answered the survey reported pain for more than 25% of the time during surgery, and more than one-fourth have sought medical attention for this pain. In addition, we found a higher rate of pain during surgery for major open surgery compared to robotic surgery. In total, 75% (54/72) of the respondents have received no formal ergonomic training, which could potentially decrease pain, health care costs, and burnout. Although ergonomic changes have continued to be implemented, such as with advanced robotic consoles, work-related pain is still prevalent.

## Supplementary material

10.2196/88848 Multimedia Appendix 1 Survey distributed to members of the Florida Urologic Society to assess prevalence and characteristics of intraoperative discomfort.

## Abbreviations

FUS: Florida Urologic Society
WRMD: work-related musculoskeletal disorder
