# Supplementary material for: Prevalence of Work-Related Pain or Discomfort Among Urologists in the State of Florida: Results From the Florida Urologic Society Task Force on Ergonomic Challenges Experienced by Its Members
Source: JMIR Hum Factors. 2026 Jun 17;13:e88848. doi: 10.2196/88848 (PMC13274963; doi:10.2196/88848)
Supplement: Multimedia Appendix 1 [file humanfactors-v13-e88848-s001.docx]

Start of Block: Default Question Block

| 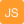 |
| --- |

Q1 **The Florida Urologist Work-Related Pain/Discomfort and Ergonomics Questionnaire**


 **In which type of urological practice do you work?**

- Teaching-based hospital with resident assistance (1)
- Teaching-based hospital without resident assistance (2)
- Community hospital (3)
- Private practice (4)

| Page Break |  |
| --- | --- |

Q2 **During the last work week, how often did you experience ache, pain, and/or discomfort in:**1

|  | Never (1) | 1 to 2 times last week (2) | 3 to 4 times last week (3) | Once every day (4) | Several times every day (5) |
| --- | --- | --- | --- | --- | --- |
| Headache (1) |  |  |  |  |  |
| Eye pain (2) |  |  |  |  |  |
| Neck (3) |  |  |  |  |  |
| Shoulder (4) |  |  |  |  |  |
| Upper arm (5) |  |  |  |  |  |
| Forearm (6) |  |  |  |  |  |
| Wrist (7) |  |  |  |  |  |
| Upper back (8) |  |  |  |  |  |
| Lower back (9) |  |  |  |  |  |
| Hip/buttocks (10) |  |  |  |  |  |
| Thigh (11) |  |  |  |  |  |
| Knee (12) |  |  |  |  |  |
| Lower leg (13) |  |  |  |  |  |
| Foot (14) |  |  |  |  |  |

| Page Break |  |
| --- | --- |

Q3 **To what do you assign your work-related pain/discomfort?**  (Select all that apply.)

- ⊗No work-related pain/discomfort (1)
- Uncomfortable position while operating (2)
- Extensive standing (3)
- Equipment hindrance (loupes, lead, gown, gloves) (4)
- Heavy tools (endoscope, tools, etc.) (5)
- Moving/positioning patients (6)
- Battering/physical abuse by patient (7)
- Other, please specify: (8) __________________________________________________

Q4 **How have you attempted to minimize your operative discomfort or pain?**  (Select all that apply.) (Rotate phone to vertical.)

- Seek medical help (1)
- Changing position (2)
- Take a break (3)
- Go slower (4)
- Adjust some aspect of the surgical field (5)
- Change height by using step (6)
- Use chair for seated surgery (7)
- Changing instruments (8)
- Switch to robotic surgery (9)
- Consider possible physical discomfort or symptoms when choosing an operative approach for an individual patient (10)
- Reduce case load (11)
- Foot wear or support stockings (12)
- Surgical floor mats (13)
- Time away from the OR (14)
- Over-the-counter medicine/analgesic (15)
- Ignore it (16)
- Other, please specify: (17) __________________________________________________
- ⊗N/A (18)

| Page Break |  |
| --- | --- |

Q5 **Which type(s) of neuromusculoskeletal disorders have you been diagnosed with that you attribute to your work as a surgeon?**  (Select all that apply.)

- Cervical disc issues (1)
- Other neck issues (2)
- Shoulder pain (tendonitis) (3)
- Other shoulder issues such as arthritis/inflammation (4)
- Rotator cuff injury (5)
- Tennis elbow (Lateral Epicondylitis) (6)
- Golfer’s elbow (Medial Epicondylitis) (7)
- Wrist/forearm tendonitis (8)
- Wrist/forearm tenosynovitis (9)
- Carpal tunnel syndrome (10)
- Prolapsed/herniated disc (11)
- Other lumbar disc issues (12)
- Knee osteoarthritis (13)
- ACL/meniscus tear/knee injury (14)
- Hip arthritis (15)
- Plantar fasciitis (16)
- Other, please specify: (17) __________________________________________________
- ⊗N/A (18)

Q6 **What type of medical help have you sought for your work-related aches, pain, or discomfort?**  (Select all that apply.)

- Prescription medicine (1)
- Injections (2)
- Massage therapy (3)
- Chiropractic (4)
- Acupuncture (5)
- Using a brace or support device (6)
- Cervical traction device (7)
- Physiotherapy (8)
- Referral to specialist (neurology, orthopedics, physical medicine, and rehabilitation, etc.) (9)
- Diagnostic studies (X-ray, CT, MRI, etc.) (10)
- Surgery (11)
- Other, please specify: (12) __________________________________________________
- ⊗N/A (13)

| Page Break |  |
| --- | --- |

Q7 **Does your work-related physical discomfort or pain affect any of the following?**  (Select all that apply.)

- Sleep (1)
- Relations with other people (2)
- Surgical posture (3)
- Balance (4)
- Concentration (5)
- Mobility (6)
- Object visualization (7)
- Patience with others (8)
- Degree of irritability (9)
- Intolerance or frustration (10)
- Willingness to teach (11)
- Stamina (12)
- Surgical speed (13)
- Tremor (14)
- Choice of type of surgery performed (ex. Open vs endoscopic vs laparoscopic vs robotic) (15)
- Other, please specify: (16) __________________________________________________
- ⊗N/A (17)

| 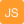 |
| --- |

Q8 **In what ways did work-related pain/discomfort affect your work?**

- I was not affected (1)
- Capable of work with minor pain/discomfort (2)
- Capable of work with moderate pain/discomfort (3)
- Capable of work with severe pain/discomfort (4)
- Had to take a leave of absence from work (5)
- Capable of clinical duties, but diminished ability to do surgery (6)
- Pain/discomfort caused you to retire at an earlier age (7)
- Other, please specify: (8) __________________________________________________

| Page Break |  |
| --- | --- |

| 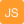 |
| --- |

Q9 **How often do you perform endoscopy in the OR in a typical week?**

- 0 cases (1)
- 1 to 5 cases (2)
- 6 to 10 cases (3)
- More than 10 cases (4)

| 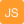 |
| --- |

Q10 **How often have you experienced work-related pain/discomfort while performing endoscopic surgery?**

- I do not experience work-related pain while performing endoscopic surgery (1)
- Up to 25% of the time (2)
- 26 to 50% of the time (3)
- 51 to 75% of the time (4)
- 76 to 100% of the time (5)
- N/A (6)

| Page Break |  |
| --- | --- |

| 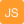 |
| --- |

Q11 **How often do you perform major open surgeries (Cystectomy, prostatectomy, nephrectomy, etc.) that take more than 3 hours in a typical week?**

- 0 cases (1)
- 1 to 2 cases (2)
- 3 to 4 cases (3)
- 5 to 6 cases (4)
- More than 6 cases (5)

| 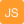 |
| --- |

Q12 **How often have you experienced work-related pain/discomfort while performing major open surgery?**

- I do not experience work-related pain while performing major open surgery (1)
- Up to 25% of the time (2)
- 26 to 50% of the time (3)
- 51 to 75% of the time (4)
- 76 to 100% of the time (5)
- N/A (6)

| Page Break |  |
| --- | --- |

| 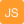 |
| --- |

Q13 **How often do you perform minor open surgeries (hydrocelectomy, IPP/AUS, sling placement, scrotal cases, etc.) in a typical week?**

- 0 cases (1)
- 1 to 2 cases (2)
- 3 to 4 cases (3)
- 5 to 6 cases (4)
- More than 6 cases (5)

| 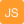 |
| --- |

Q14 **How often have you experienced work-related pain/discomfort while performing minor open surgery?**

- I do not experience work-related pain while performing minor open surgery (1)
- Up to 25% of the time (2)
- 26 to 50% of the time (3)
- 51 to 75% of the time (4)
- 76 to 100% of the time (5)
- N/A (6)

| Page Break |  |
| --- | --- |

| 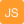 |
| --- |

Q15 **How often do you perform robotic surgery in a typical week?**

- 0 cases (1)
- 1 to 3 cases (2)
- 4 to 6 cases (3)
- 7 to 9 cases (4)
- 10 or more cases (5)

| 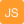 |
| --- |

Q16 **How often have you experienced work-related pain/discomfort while performing robotic surgery?**

- I do not experience work-related pain while performing robotic surgery (1)
- Up to 25% of the time (2)
- 26 to 50% of the time (3)
- 51 to 75% of the time (4)
- 76 to 100% of the time (5)
- N/A (6)

| Page Break |  |
| --- | --- |

| 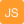 |
| --- |

Q17 **Have you ever suggested alternative treatment options or modalities to patients for fear of work-related pain/discomfort?**

- Yes (1)
- No (2)
- Prefer not to answer (3)

| 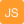 |
| --- |

Q18 **How many days of work have you missed in the last year due to work-related pain/discomfort?**

- 0 days (1)
- 1 to 3 days (2)
- 4 to 6 days (3)
- 7 to 10 days (4)
- 10 or more days (5)
- Prefer not to answer (6)

| 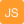 |
| --- |

Q19 **Have you ever received ergonomic training?**

- Yes (1)
- No (2)
- Prefer not to answer (3)

| 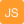 |
| --- |

Q20 **Would you be interested in receiving official ergonomic surgical training?**

- Yes (1)
- No (2)

| Page Break |  |
| --- | --- |

| 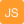 |
| --- |

Q21 **How many years have you been a urologist?**

- Urology resident year 1 to 5 (1)
- 1 to 5 years into practice (2)
- 6 to 10 years into practice (3)
- 11 to 15 years into practice (4)
- 16 to 20 years into practice (5)
- 20 or more years into practice (6)

| 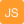 |
| --- |

Q22 **How do you identify?**

- Female (1)
- Male (2)
- Another description, please specify: (3) __________________________________________________
- I prefer not to answer (4)

| Page Break |  |
| --- | --- |

| 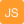 |
| --- |

Q23 **How tall are you?**

- Less than 5’0" (1)
- 5’0" to 5’4’’ (2)
- 5’5’’ to 5’8’’ (3)
- 5’9 to 6’0" (4)
- More than 6’0" (5)
- Prefer not to answer (6)

| 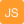 |
| --- |

Q24 **What is your estimated weight?**

- Below 100 lbs. (1)
- 100 to 150 lbs. (2)
- 151 to 200 lbs. (3)
- 201 to 250 lbs. (4)
- 251 to 300 lbs. (5)
- 301 to 350 lbs. (6)
- 351 lbs. or more (7)
- Prefer not to answer (8)

End of Block: Default Question Block

Start of Block: Submit

Q25 Reference
 1Adapted from Nordic musculoskeletal questionnaire

   THANK YOU FOR COMPLETING THE SURVEY!

 **Please click SUBMIT to record your answers.**

End of Block: Submit
